# Identification and characterization of a FOXA2-regulated transcriptional enhancer at a type 2 diabetes intronic locus that controls *GCKR* expression in liver cells

**DOI:** 10.1186/s13073-017-0453-x

**Published:** 2017-07-06

**Authors:** Maykel López Rodríguez, Dorota Kaminska, Kati Lappalainen, Jussi Pihlajamäki, Minna U. Kaikkonen, Markku Laakso

**Affiliations:** 1 Institute of Clinical Medicine, Internal Medicine, University of Eastern Finland, Yliopistonranta 1 C, 70211 Kuopio, Finland; 2 Institute of Public Health and Clinical Nutrition, University of Eastern Finland, P.O. Box 1627, FI-70211 Kuopio, Finland; 3 Department of Human Genetics, David Geffen School of Medicine at UCLA, Los Angeles, California USA; 4 A.I. Virtanen Institute for Molecular Sciences, Department of Biotechnology and Molecular Medicine, University of Eastern Finland, P.O. Box 1627, 70211 Kuopio, Finland; 5 Institute of Public Health and Clinical Nutrition, University of Eastern Finland, Kuopio campus, P.O. Box 1627, FI-70211 Kuopio, Finland; 6 Clinical Nutrition and Obesity Center, Kuopio University Hospital, P.O. Box 100, FI 70029 KYS Kuopio, Finland; 7 Department of Medicine, Kuopio University Hospital, P.O. Box 100, FI 70029 KYS Kuopio, Finland

**Keywords:** rs780094, rs780095, rs780096, GCKR, Transcriptional enhancer, FOXA2, Type 2 diabetes

## Abstract

**Background:**

Genome-wide association studies (GWAS) have identified more than 100 genetic loci associated with type 2 diabetes (T2D). However, the underlying biological mechanisms for many of these associations remain unknown. GWAS signals close to the glucokinase regulatory protein gene (*GCKR*) have been reported for lipid and glucose metabolism traits and the risk of T2D. We investigated the regulatory function of an intronic locus at *GCKR* represented by the lead single nucleotide polymorphism (SNP) rs780094.

**Methods:**

We used ENCODE project histone modification and transcription factor binding data to determine the regulatory features of a *GCKR* intronic locus formed by the high linkage disequilibrium rs780094(C/T), rs780095(G/A), and rs780096(G/C) SNPs. Characterization of the transcriptional activity of this region was assessed by luciferase reporter assays in HepG2 cells and mouse primary hepatocytes. ChIP-qPCR was used to determine the levels of haplotype specific transcription factor binding and histone marks. A CRISPR-dCas9 transcriptional activator system and qPCR were used to activate the locus and measure *GCKR* expression, respectively. Differential haplotype expression was measured from human liver biopsies.

**Results:**

The ENCODE data suggest the existence of a liver-specific intragenic enhancer at the locus represented by s780094. We observed that FOXA2 increased the transcriptional activity of this region in a haplotype specific way (CGG > TAC; rs780094, rs780095, and rs780096). In addition, the CGG haplotype showed higher binding to FOXA2 and higher levels of the H3K27Ac histone mark. The epigenetic activation of this locus increased the expression of endogenous *GCKR* in HepG2 cells, confirming that *GCKR* is the direct target gene of the enhancer. Finally, we confirmed that the CGG haplotype exhibits higher levels of transcription in human liver.

**Conclusions:**

Our results demonstrate the existence of a liver-specific FOXA2-regulated transcriptional enhancer at an intronic T2D locus represented by rs780094, rs780095, and rs780096 SNPs that increases *GCKR* expression. Differential haplotype regulation suggests the existence of *cis* regulatory effects that may contribute to the associated traits at this locus.

**Electronic supplementary material:**

The online version of this article (doi:10.1186/s13073-017-0453-x) contains supplementary material, which is available to authorized users.

## Background

Type 2 diabetes (T2D; MIM 125853) develops as a consequence of complex interactions between genetic and environmental/lifestyle factors. Genome-wide association studies (GWAS) have identified more than 100 common single nucleotide polymorphisms (SNPs) associated with T2D and glycemic traits [1]. Although some association signals are due to coding variants, the majority of signals do not map to protein coding sequence, which suggests that SNPs influence gene regulation.

The glucokinase regulatory protein gene (*GCKR*; MIM 613463) encodes for glucokinase regulatory protein (GKRP; NP_001477), the main allosteric inhibitor of glucokinase (HK4; P35557.1; GCK hereafter), and is a T2D-associated locus. GKRP inhibits GCK by a direct effect on enzyme kinetics or by sequestering GCK in the nucleus, where GCK is unable to facilitate glycolysis [2]. This inhibitory mechanism operates as a negative feedback loop in the control of glucose disposal by the liver. In the fasting state, GCK is bound to GKRP and retained in the nucleus. In the fed state, the increase in glucose concentrations induces the dissociation of GCK from GKRP, which activates GCK and facilitates its release into the cytoplasm [2, 3]. The subsequent increase in fructose-6 phosphate, attributable to glycolysis, results in the re-formation of the GKRP–GCK complex and enzyme inhibition [2, 3]. Despite the inhibitory effect of GKRP on GCK, this nuclear sequestration during fasting is proposed to protect GCK from degradation, which in turn guarantees an effective pool of the enzyme to be released into the cytoplasm in response to glucose [4]. Given the major role of hepatic GCK in glucose metabolism in the liver, disturbances in GKRP–GCK regulation at the transcriptional or post-transcriptional level may directly affect glucose metabolism and produce subsequent changes in other metabolic traits.

*GCKR* lies in a large region of linkage disequilibrium (LD) on chromosome 2, spanning about 417 kb, 16 genes, and several correlated variants [5]. Genetic fine-mapping of this region has localized a GWAS signal to *GCKR* rather than to other genes in the LD block [5]. These studies also identified the nonsynonymous rs1260326 SNP (C/T, P446L substitution) as the strongest signal for fasting blood glucose and total triglycerides in this region [5]. Functional studies on this variant demonstrate that the P446L amino acid substitution results in lower GCK sequestration capacity and impaired response to fructose-6-P [6, 7], which is thought to affect glucose levels indirectly by affecting the cytoplasmic availability and activity of GCK [6, 7]. Thus, rs1260326 has been established as a functional SNP which is likely to have a causal relationship with GCKR-related traits.

Despite the functional evidence that the rs1260326 variant impacts on both the kinetics and cellular localization of GKRP, the high LD in the region warrants the investigation of other variants which could contribute to molecular mechanisms associated with multiple traits. One such variant is an intronic SNP, rs780094, which was originally identified to be associated with fasting serum triacyglycerol, insulinemia, and the risk of T2D [8]. As expected for SNPs in high LD, rs780094 and rs1260326 (r^2^ = 0.94) overlap in their phenotypical associations [9]. Thus, their independent effects cannot be accurately assessed based on association analysis. While a molecular mechanism has been elucidated for the P446L variant [6, 7], no functional role has been reported for rs780094. Given its location at the non-coding region, we hypothesized that this locus could regulate *GCKR* transcription. Another two SNPs, rs780095 and rs780096, are in high LD (r^2^ = 0.86) and are located very close (132 and 165 bp, respectively) to rs780094. Therefore, it is likely that these variants overlap in their functional roles and thus their haplotype combinations may influence the metabolic susceptibility.

In the present study we demonstrate that the locus containing rs780094 functions as a transcriptional enhancer that is activated by the Forkhead box protein A2 (FOXA2; Q9Y261) transcription factor (TF) in a haplotype specific fashion. We show that epigenetic activation of the enhancer induces *GCKR* expression and that *GCKR* is a direct target of this regulatory region. In addition, we show an association between haplotype expression and the levels of free fatty acids (FFAs) in human liver biopsies. Our results suggest a novel mechanism for the regulation of *GCKR* expression that could have a combined effect with the P446L variant on the phenotypical associations at the *GCKR* locus.

## Methods

### Culture and stimulation of HepG2

HepG2 cells (ATCC, HB-8065) were cultured in Dulbecco’s modified Eagle medium (DMEM; 4.5 g/L glucose, 2 mM L-glutamine, 100 U/ml penicillin, 100 μg/ml streptomycin; LONZA) supplemented with 10% fetal bovine serum (FBS; GIBCO). Cells were seeded at 0.2–0.3 × 10^6^ cells/ml in the appropriate vessel depending on the assay performed. For insulin stimulation in ChiP-qPCR experiments, the cells were serum-starved overnight and insulin (human recombinant insulin, Sigma-Aldrich; 100 nM) was added for 15 min before crosslinking. The cells were transfected using Lipofectamine 3000 (ThermoFisher) according to the manufacturer’s indications. The transfection conditions were optimized before conducting the over-expression experiments.

### Preparation and stimulation of mouse primary hepatocytes

Mouse primary hepatocytes (MPHs) were prepared from 7–9-week-old C57BL male mice livers as previously described [10]. Briefly, mice were anesthetized before dissection and the skin was opened by making a longitudinal middle ventral incision followed by two transversal incisions below the level of the liver. The vena cava superior was cannulated with a cannulation system (Fine Science Tools) coupled to a peristaltic pump (BioRad), a ligature was placed around the vena cava inferior, and the vena porta was cut open. Perfusion was performed with 250 ml of pre-perfusion solution (0.04% NaHCO_3_, 10 mM HEPES, 0.5 mM EDTA in HBSS; pH adjusted to 7.4) followed by 35 ml of perfusion solution (10 mM HEPES, 80 U/ml collagenase [Worthington] in William’s E medium [Sigma-Aldrich]; pH adjusted to 7.4). Both the pre-perfusion and the perfusion solutions were pre-warmed to 37 °C and circulated through an oxygen atmosphere and a 37 °C water bath before reaching the liver at 3 ml/min. After the perfusion, the liver was removed and placed on a petri dish containing a sufficient amount of plating medium (William’s E medium with 20 ng/ml dexamethasone [Sigma-Aldrich], ITS [5 mg/l insulin/5 mg/l transferrin/5 μg/l sodium selenite; Sigma-Aldrich], 10 μg/ml gentamicin [Sigma-Aldrich], and 10% FBS) and torn into pieces with sharp forceps to extract the cells. The cell suspension was filtrated through a cell strainer (40 μm; Falcon) and centrifuged at 50 × g for 2 min. Cells were seeded at 0.5 × 10^6^ cells/ ml on collagen-coated 96-well plates. Five hours after plating, medium was changed to maintenance medium (seeding medium without FBS), and the following day cells were transfected with Targefect F1 plus Virofect enhancer transfection reagents (Targeting Systems) according to the manufacturer’s indications. DNA constructs were expressed for 24 h in basal William’s E medium with 20 ng/ml dexamethasone without or with 100 nM of insulin. All animal experiments in this study were approved by the National Experimental Animal Board of Finland.

### Luciferase reporter transcriptional assays

**Fig. 1:**
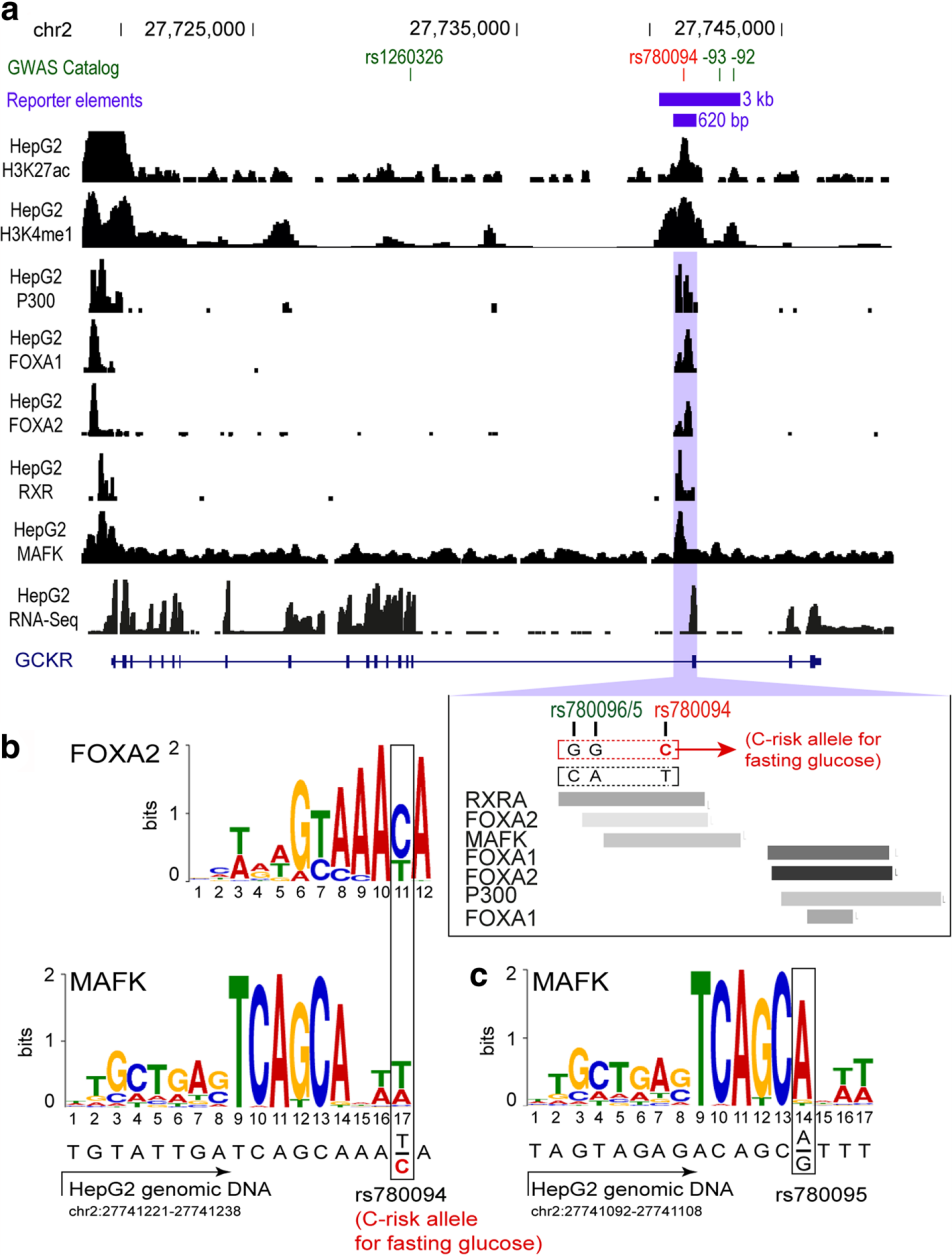
rs780094, rs780095, and rs780096 *GCKR* SNPs reside in a liver-specific enhancer region. **a** UCSC genome browser image of *GCKR* in liver-derived HepG2 cells. Normalized ChIP-Seq counts are shown for H3K27ac and H3K4me1 enhancer marks and P300, RXRA, FOXA2, and MAFK TF binding. The GWAS catalog SNPs and reporter elements used in the transcriptional activity assays are depicted above the figure. The TF binding sites located within the 638-bp region around rs780094 are shown in close-up in the *insert* below the image. **b** The location of FOXA2 and MAFK consensus motifs in HepG2 cells overlapping rs780094 as defined by Factorbook [54]. Position weight matrix (PWM) modeling shows that rs780094 is likely to disrupt the binding of MAFK, whereas both alleles are predicted to allow binding of FOXA2. **c** The location of MAFK consensus motifs overlapping rs780095. The disruption of MAFK motif is predicted by PWM modeling

**Fig. 2:**
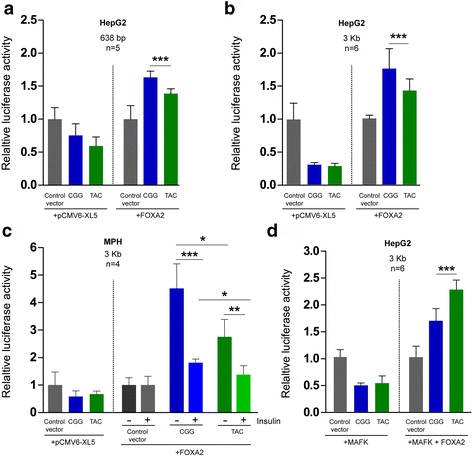
Haplotype-specific transcriptional activity. Luciferase reporter assays. Firefly luciferase activity was normalized to the *Renilla* luciferase signal and the data are shown as proportions of control vector pGL4.10-TK. **a** HepG2 cells with 638-bp constructs. *Right panel*: FOXA2 induced a haplotype-specific transcriptional activity (CGG > TAC) compared to the control pGL4.10-TK. *Error bars* represent the standard deviation of five experiments (*n* = 5) with three, three, eight, four, and four technical replicates (***p ≤ 0.005; two-tailed *t*-test). **b** HepG2 cells with 3-kb constructs. *Right panel*: FOXA2 induced the transcriptional activity of the inserts in a haplotype-specific way (CGG > TAC) compared to the control pGL4.10-TK. *Error bars* represent the standard deviation of six experiments (*n* = 6) with four, four, eight, eight, four, and four technical replicates (***p ≤ 0.005; two-tailed *t*-test). **c** MPHs with 3-kb constructs. *Right panel*: insulin reduced FOXA2-induced transcriptional activity in MPHs. *Error bars* represent standard deviation of four biological replicates corresponding to four livers (*n* = 4) with two technical replicates each (*p ≤ 0.05; **p ≤ 0.01; ***p ≤ 0.005; *t*-test for comparison between haplotypes within treatment; one-way ANOVA for comparisons between the treatments). **a**–**c** *Left panels*: in co-transfection with the pCMV6-XL5 vector without a TF insert, none of the haplotypes show transcriptional activity with respect to the control plasmid pGL4.10-TK. **d** HepG2 cells with 3-kb constructs. *Left panel*: in co-transfection with MAFK and pCMV6-XL5, none of the haplotypes show transcriptional activity. *Right panel*: co-transfection of FOXA2 and MAFK induced transcriptional activity from both 3-kb regions but inverted the haplotype bias compared to FOXA2 alone (TAC > CGG). *Error bars* represent standard deviation of six experiments (*n* = 6) with six, six, six, six, four, and four technical replicates (***p ≤ 0.005; two-tailed *t*-test)

DNA fragments containing the rs780094-rs780095-rs780096 region (Fig. 1a) were amplified by PCR from human homozygous genomic DNA (gDNA) selected from the METSIM study [11] with Phusion polymerase and specific primers (Additional file 1: Table S1). We selected the two most common haplotypes formed by these SNPs in the METSIM population [11] (CGG; TAC; rs780094-rs780095-rs780096; Additional file 2: Datasets). Amplified DNA was subcloned into BamHI and SalI restriction enzyme sites downstream of the firefly luciferase gene in a pGL4.10 vector (Promega) modified with a minimal TK (thymidine kinase) promoter as previously described (pGL4.10-TK) [12]. In total, four DNA inserts were amplified, two 638-bp inserts (one per haplotype) and two 3-kb inserts (one per haplotye). The luciferase constructs prepared with the inserts were verified by sequencing. HepG2 cells were transfected with 100 ng of plasmid DNA on 96-well plates. The luciferase constructs or the control vector pGL4.10-TK were co-transfected with pGL4.75 plasmid (Promega) encoding the luciferase gene hRluc (*Renilla reniformis*) and plasmids expressing human FOXA2, FOXA1, RXRA, or MAFK or the control vector pCMV6-XL5 without TF insert (SC122913; SC118299; SC118696; SC108256; pCMV6-XL5; OriGene) (molar ratios: 1:10, pGL4.75 to PGL4.10-TK; 1:1, luciferase constructs or PGL4.10-TK to TF plasmids or pCMV6-XL5; 1:1 FOXA2 to MAFK). The luciferase activity was measured 48 h post-transfection using the Dual-Glo Luciferase Assay System (Promega) in the Cytation 3 Multi-Mode Reader (BioTek). MPH were transfected with 60 ng of plasmid DNA and 24 h post-transfection luciferase activity was measured with the Dual Luciferase Assay System (Promega) in a Fluostar OPTIMA plate reader (BMG LABTECH). In all cases, the firefly luciferase activity was normalized to the *Renilla* signal and reported as proportions relative to the control vector. In HepG2 cells, we performed five or six independent experiments with three or more technical replicates (Fig. 2a, b, d; Additional file 2: Datasets). For the experiments in MPHs, four biological replicates corresponding to four livers were used (with two technical replicates each; Fig. 2c).

### Genotyping of HepG2 cells and human samples by sequencing

gDNA from HepG2 cells or blood (from human donors) was extracted using the PureLink genomic DNA Mini Kit (Invitrogen). A 3-kb region from gDNA was amplified by PCR with Phusion High-Fidelity DNA polymerase (ThermoFisher) and specific primers (Additional file 1: Table S1) in a PRISM 2720 thermal cycler (Applied Biosystems). PCR amplicons were purified using the ChIP DNA Clean and Concentrator Kit (Zymo Research). For sequencing, the samples were prepared using the Nextera XT DNA Library Preparation Kit (Illumina) and ran on a MiSeq desktop sequencer (Ilumina). The sequence reads were aligned to the human genome (hg19) using the Burrows–Wheeler Aligner (BWA) [13], and the allelic distributions were obtained with the Integrative Genomics Viewer (IGV) [14, 15].

### ENCODE ChIP-seq data analysis

The processed data for HepG2 ChIP-Seq (bam and bigwig files) were downloaded from the ENCODE database under accession numbers GSM733743, GSM803499, GSM803461, GSM803403, GSM803452, GSM935610, GSM803432, GSM733693, GSM935610, and GSM935305. The allele-specific ChIP-Seq data were visually quantified from bam files using the IGV [14, 15]. These values were normalized to account for the allelic imbalance at rs780094 (C = 2XT).

### ChIP-qPCR

ChIP experiments were performed in HepG2 cells with minor changes from a previously published protocol [16]. Briefly, the cells were crosslinked in 1% formaldehyde (Sigma-Aldrich) for 10 min at room temperature and crosslinking was stopped with 0.125 M of glycine. Chromatin was fragmented by sonication using Bioruptor UCD-300 (Diagenode). Antibodies (FOXA2, RRID:AB_2262810; H3K27Ac, RRID:AB_2118291; 1–3 μg per ChIP reaction) were coupled to magnetic beads (Millipore) overnight at 4 °C. For immunoprecipitation, a 100-μl aliquot of sonicated chromatin was incubated overnight with antibody-coupled magnetic beads, washed, and eluted by incubation at 65 °C for 1 h with brief vortexing every 15 min. To completely revert crosslinking, the eluted chromatin was incubated overnight at 65 °C with proteinase K (ThermoFisher). The fragmented chromatin was purified using a ChIP DNA Clean and Concentrator Kit (Zymo Research). qPCR was conducted using a custom TaqMan SNP Genotyping Assay for rs780094 (ThermoFisher). This assay uses allele-specific probes coupled to different fluorescent dyes in the same reaction mix for the quantitative detection of the alleles in a single sample. The rs780094 assay amplifies a 70-bp region around this SNP (Additional file 1: Table S1). We used the *dd*Ct method to calculate the haplotype-specific enrichment for both input and immunoprecipitated chromatin over an intronic region of *GRB10* (rs6943153; MIM 601523) for which no TF binding or H3K27Ac histone marks have been reported. Fold enrichment was determined by dividing the enrichment of the immunoprecipitated chromatin by the enrichment of the respective input. FOXA2 and H3K27Ac ChIP-qPCR experiments were performed in triplicate (three technical replicates in each experiment for the determination of basal H3K27Ac levels; two, two, and three technical replicates for FOXA2 binding experiments; three, two, and three technical replicates for H3K27Ac in the CRISPR assays).

### CRISPR-dCas9-VPR-mediated transcriptional activation

To determine the direct effect of the enhancer on *GCKR* expression we used a CRISPR-dCas9-derived transcriptional activator system [17, 18]. This system comprises two plasmids, one expressing a nuclease-null Cas9 (dCas9) fused in tandem with three transcriptional activation domains (VP64, p65, and Rta [VPR]) [17], and the second one expressing the gRNA that directs the dCas9-VPR activator to the target site [18]. The gRNAs were designed using an online tool [19] and two gRNAs sequences, located adjacent to rs780094 in a SNP-free region, were selected for cloning. Briefly, forward and reverse primers were synthesized comprising the gRNA sequences provided by the online designer (Additional file 1: Table S1). The primer pairs were phosphorylated using T4 Polynucleotide Kinase (ThermoFisher) and annealed in the thermal cycler with the following program: 37 °C for 30 min; 95 °C for 5 min; ramp down to 25 °C at 5 °C /min. Double-stranded oligos were subcloned into pSPgRNA plasmid (a gift from Charles Gersbach; Addgene plasmid # 47108) digested with BbsI (ThermoFisher). The identity of the gRNA constructs was verified by sequencing. HepG2 cells were co-transfected with the activator VPR and each of the gRNA constructs or an empty gRNA vector in a 1:1 mass ratio. The constructs were expressed for 48 h and thereafter total RNA was extracted using the RNeasy Mini Kit (Qiagen). cDNA was prepared with random hexamer primers using a High Capacity cDNA Reverse Transcription Kit (Applied Biosystems, Foster City, CA, USA) according to the manufacturer’s instructions. *GCKR* mRNA and *RPLP0* mRNA (housekeeping gene; MIM 180510) expression were measured by qPCR using specific TaqMan gene expression assays (ThermoFisher) in a 7500 Real-Time PCR System (Applied Biosystems). The relative gene expression was calculated using the relative *dd*Ct-quantification. Four independent experiments were performed (with three, four, four, and three technical replicates, respectively).

### Haplotype-specific expression in human liver biopsies

A total of 304 severely obese individuals participated in the ongoing KOBS study [20]. All subjects underwent Roux-en-Y gastric bypass surgery at the Kuopio University Hospital (Kuopio, Finland). The study protocol was approved by the Ethics Committee of Northern Savo Hospital District (Finland) and carried out in accordance with the Helsinki Declaration. Informed written consent was obtained from all participants. Liver tissue biopsies used in our study were collected during the operation. A total of 132 subjects heterozygous for rs780094, rs780095, and rs780096 were identified and included in this study (Additional file 2: Datasets). Total RNA from human liver tissue biopsies was extracted with the miRNeasy Mini Kit (Qiagen) and the cDNA was prepared as described above. Haplotype-specific expression was determined with the TaqMan SNP genotyping assay for rs780094, as previously reported [21]. The expression was quantified using the *dd*Ct method and the relative expression between the haplotypes was calculated. The haplotype expressions were compared using a Mann–Whitney *U* test, and the associations between haplotype expression and metabolic traits were assessed by a Spearman’s rank correlation test with Bonferroni correction.

### Analytical methods used in the human study

Plasma glucose, insulin, serum lipids and lipoproteins (total cholesterol, HDL cholesterol, and triglycerides), and FFAs were measured from fasting venous blood samples. Plasma glucose was measured by enzymatic hexokinase photometric assay (Konelab Systems Reagents; ThermoFisher). Plasma insulin was determined by immunoassay (ADVIA Centaur Insulin IRI, Siemens Medical Solutions Diagnostics). An oral glucose tolerance test (OGTT) was performed after a 12-h fasting period. Serum FFAs were assayed with an enzymatic colorimetric method (Wako NEFA C test kit; Wako Chemicals). Glucose, insulin, and FFA levels were determined at 0, 30, and 120 min with an OGTT.

### Statistical analysis

**Fig. 3:**
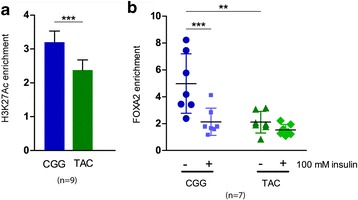
Haplotype-specific H3K27Ac levels and FOXA2 binding by ChIP-qPCR. Chromatin was immunoprecipitated and H3K27Ac levels and FOXA2 binding was determined by qPCR using a custom TaqMan SNP Genotyping Assay for rs780094. The data represent the haplotype-specific enrichments over the input, normalized to a region of *GRB10* with no H3K27Ac marks or TF binding. **a** Basal H3K27Ac enrichment (three experiments with three replicates each [n = 9]). **b** FOXA2 enrichment in FOXA2-transfected HepG2 cells (three experiments with two, two, and three technical replicates, respectively; n = 7). Insulin (100 nM) reduced the binding of FOXA2 to the CGG haplotype. In both panels, *error bars* represent standard deviation of all technical replicates. The *asterisks* depict statistical significance (**p ≤ 0.01; ***p ≤ 0.005; two-tailed *t*-test for comparison between the haplotypes; one-way ANOVA for comparisons between treatments (**b**))

**Fig. 4:**
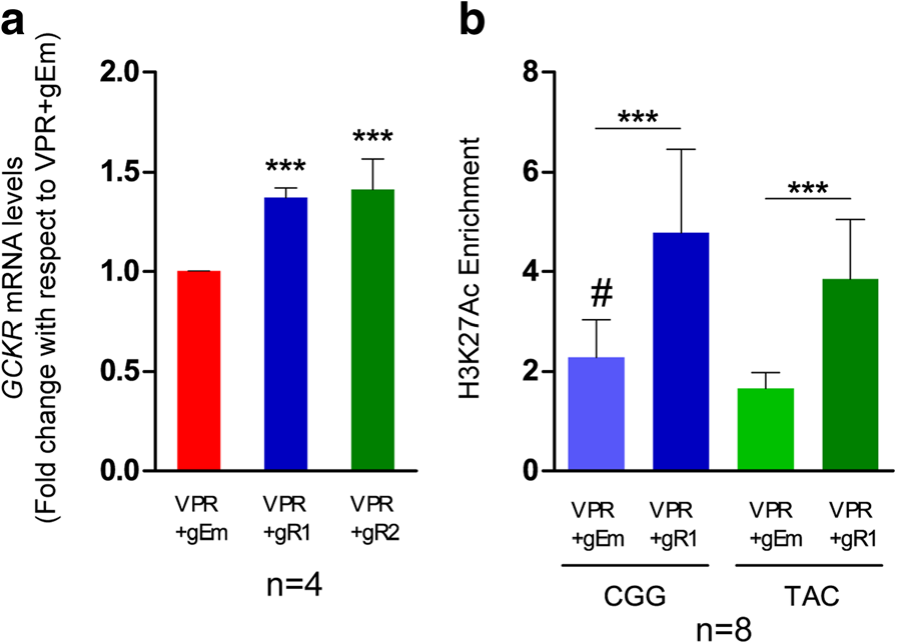
Activation of the enhancer by CRISPR-dCas9-VPR induces *GCKR* expression. HepG2 cells were co-transfected with the VPR activator plasmid and the guide RNA plasmids targeting (*gR1* or *gR2*) or not targeting (*gEm*) the enhancer locus. **a** Total *GCKR* mRNA levels determined by qPCR with a *GCKR* Taqman gene expression assay. The data represent *GCKR* mRNA levels relative to non-targeting VPR + gEm, normalized to housekeeping gene *RPLP0. Error bars* represent standard deviation of four independent experiments (n = 4) with three, four, four, and three technical replicates, respectively. **b** Haplotype-specific enrichment of H3K27Ac (enrichment over input normalized to a region of *GRB10* with no TF binding or active histone marks [rs6943153]) determined by ChIP-qPCR using the custom Taqman SNP Genotyping Assay for rs780094 (three independent experiments with three, two, and three technical replicates, respectively). *Error bars* represent the standard deviation between technical replicates (n = 8). *Asterisks* and the *hash symbol* depict statistical significance (***p ≤ 0.005; #*p* ≤ 0.5 and refers to comparison between haplotypes upon VPR + gEm; **a** one-way ANOVA with Tukey’s post-hoc test; **b** two-tailed *t*-test for comparisons between two groups)

**Fig. 5:**
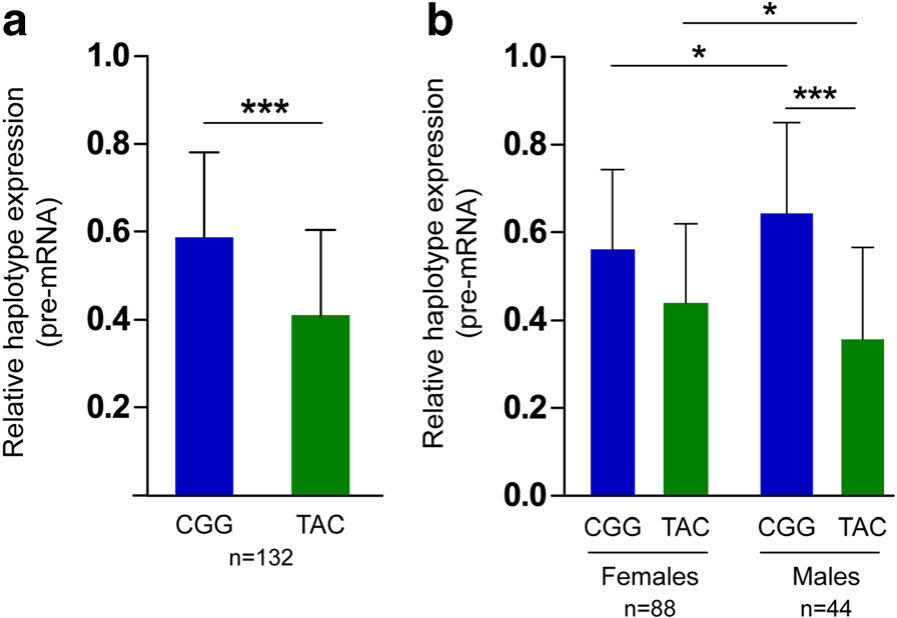
Haplotype-specific expression in human liver biopsies. Haplotype-specific levels of pre-mRNA expression determined with the TaqMan genotyping assay for rs780094. **a** Expression of the CGG and TAC haplotypes in human liver biopsies from 132 individuals heterozygous for rs780094, rs780095, and rs780096. **b** Stratification by sex. The data represent the mean expression of each haplotype relative to the other. *Error bars* represent the standard deviation and the *asterisks* depict statistical significance (*p ≤ 0.05; ***p ≤ 0.005; Mann–Whitney *U* test)

In the luciferase assays, the technical replicates of each experiment (Fig. 2) were averaged and the mean value of the haplotypes per experiment was generated. We used the two-tailed *t*-test to compare the haplotypes in the same treatment, and one-way ANOVA to compare the haplotypes between the different treatments (insulin versus no insulin). Basal H3K27Ac and FOXA2 ChIP-qPCR data (Fig. 3a, b) were analyzed using technical replicates. Comparisons between the haplotypes of the same treatment were carried out with a two-tailed *t*-test and a one-way ANOVA was used to compare the same haplotype between the treatments (Fig. 3b). For the comparisons of *GCKR* gene expression in the CRISPR activator experiments (Fig. 4a) we used one-way ANOVA with a Tukey’s post hoc test with the dCt values. The H3K27Ac ChIP-qPCR data (Fig. 4b) were compared using the enrichment values of technical replicates with a two-tailed *t*-test (comparison of two groups). No comparison between the haplotypes was performed for gR1 + VPR. The haplotype expression data from human liver biopsies (Fig. 5) were compared with a Mann–Whitney *U* test. A Spearman’s rank correlation test with Bonferroni correction was used to evaluate the association between haplotype expression and metabolic traits. In all cases, the number of biological and technical replicates per experiment is specified in the relevant section and in the corresponding figure legend.

## Results

### Genomic and regulatory features of the rs780094 locus

We investigated the chromatin features of the region containing rs780094, rs780095, and rs780096 by exploring the ENCODE Project data for histone modifications and TF binding [22]. These SNPs are in high LD (r^2^ = 0.86) and located very close to each other (Fig. 1a). The data from the Roadmap Epigenomic Projects [23] suggest a high liver-specificity of this regulatory locus (Additional file 3), in line with the key role of the liver in contributing to the metabolic traits associated with rs780094. Therefore, we focused our analysis on the human hepatocellular carcinoma (HepG2) cell line. In these cells, the variants reside in a region enriched for the active, enhancer-associated H3K4me1 and H3K27ac histone marks (Fig. 1a). In addition, ChIP-seq data for TFs showed the binding of FOXA2, RXRA, and MAFK TFs to this locus, which are also bound to the *GCKR* promoter (Fig. 1a). An adjacent second peak for FOXA2, along with peaks for FOXA1 and p300, are located around 250 bp 3′ of rs780094 (Fig. 1a). According to the 1000 Genomes Project data [24], rs780094 is also in strong LD with the common variants rs1260326 (r^2^ = 0.91) and rs780093 (r^2^ = 0.98), which, however, reside outside the potential enhancer. Moreover, only the region containing rs780094, rs780095, and rs780096 has been found to bind TFs. Position weight matrix (PWM) modeling for rs780094, rs780095, and rs780096 [25] showed that rs780094 overlaps a FOXA2 motif, which is not expected to affect binding (Fig. 1b). In contrast, both rs780094 and rs780095 affect the MAFK motif, which is predicted to result in decreased binding (Fig. 1b, c). This suggests that differential binding of MAFK and possibly other factors could alter the enhancer activity of this locus. Altogether, the histone modification and TF binding data for the locus containing rs780094, rs780095, and rs780096 suggest the existence of a highly liver-specific regulatory region consisting of two adjacent TF binding loci that could act together as a transcriptional enhancer.

### Haplotype-specific transcriptional activity of a locus defined by rs780094, rs780095, and rs780096

To investigate the transcriptional activity of the region defined by rs780094, rs780095, and rs780096 and its relationship to the TFs, we performed luciferase reporter assays in HepG2 cells and MPHs. We cloned two DNA inserts representing the two most common haplotypes formed by these variants in the METSIM study [11] (CGG/TAC; rs780094, rs780095, rs780096; Additional file 2: Datasets) into a luciferase vector with a minimal promoter: a 638-bp insert containing rs780094, rs780095, and rs780096 and both the 5′ and 3′ TF peaks; and a 3-kb insert spanning a larger region 3′ and 5′ from rs780094 (Fig. 1a). The sequences of the 638-bp inserts differ only in rs780094 (C/T), rs780095 (G/A), and rs780096 (G/C), while the 3-kb inserts differ also in the rs780093 SNP (C/T), which, however, resides outside the potential regulatory region.

None of the inserts increased luciferase transcription compared to the control vector pGL4.10-TK when transfected alone in HepG2 cells (data not shown). This may indicate insufficiency of the endogenous TF pool to activate the luciferase constructs transfected in excess to the cells, rather than a lack of enhancer activity. To test this, we co-transfected the 638-bp constructs with either the empty vector pCMV6-XL5 or plasmids expressing the TFs that bind to this region according to the ENCODE project data (FOXA2, FOXA1, RXRA, or MAFK). When co-transfected with pCMV6-XL5, none of the haplotypes increased luciferase activity compared to the control vector pGL4.10-TK (Fig. 1a, left panel). These results are consistent with the lack of transcriptional activity when the constructs were transfected alone. Of all TFs tested, only FOXA2 increased luciferase transcription compared to the control vector (Fig. 2a, right panel; Additional file 1: Figure S1). Moreover, the haplotypes showed different transcriptional activity (CGG > TAC; 15%; *p* = 0.0003). Similarly, FOXA2 also increased the transcriptional activity of the 3-kb region in a haplotype-specific way (Fig. 2b, right panel; CGG > TAC; 19%, *p* = 0.003), while no luciferase activity was observed in cotransfection with the pCMV6-XL5 vector (Fig 2b, left panel). The magnitude of the haplotype differences as well as the overall activating effect were modest, which may be explained, at least in part, because the enhancer is taken out of its natural context for the reporter assays [26]. Yet, these results suggest the existence of transcriptional activity for this locus and a regulatory bias between the haplotypes.

Insulin has been reported to inactivate FOXA2 [27–29]. Therefore, we used insulin stimulation to investigate whether the effects on luciferase transcription were FOXA2-specific. As high FOXA2 expression is obtained only 24 h after transfection, our experiment requires long exposure to insulin. However, HepG2 cells become insulin resistant when cultured in high glucose and high insulin media for 24 h or longer [30–32]. Therefore, we opted for MPHs as a physiological model for chronic insulin response. Consistent with the results in HepG2 cells (Fig 2b), FOXA2 increased the transcriptional activity for both haplotypes compared to the control (Fig. 2c, right panel). In untreated cells, the CGG haplotype showed higher transcriptional activity than the TAC haplotype (difference 64%, *p* = 0.02). As expected, insulin reduced FOXA2-dependent transcriptional activity similarly for both haplotypes (a 60% reduction for CGG [*p* = 0.001] and a 50% reduction for TAC [*p* = 0.01] (difference between the haplotypes *p* = 0.05; Fig. 2c, right panel). This similar responsiveness to insulin between the haplotypes can be explained by the fact that both of them, although to different extents, are activated by FOXA2. Similarly to the results in HepG2 cells (Fig. 2b), the 3-kb constructs did not show transcriptional activity in co-transfection with the pCMV6-XL5 control vector (Fig 2c, left panel).

Although MAFK alone was not sufficient to activate reported gene expression (Additional file 1: Figure S1), the PWM for rs780094 and rs780095 showed that the alternative variants rs780094-C and rs780095-G are likely to disrupt the motifs for MAFK (Fig. 1b, c). Therefore, to investigate the relationship between FOXA2 and MAFK in the transcriptional activity of this locus, we co-transfected each of the 3-kb inserts with MAFK and pCMV6-XL5 or FOXA2 in HepG2 cells. Consistent with the results from the 638-bp inserts (Additional file 1: Figure S1), MAFK alone did not increase luciferase transcription from the 3-kb constructs (MAFK + pCMV6-XL5; Fig. 2d, left panel). However, co-transfection of MAFK and FOXA2 produced effective transcriptional activity for both haplotypes with respect to the control vector (Fig. 2d, right panel). Importantly, in contrast to FOXA2 overexpression, the co-transfection with MAFK led to the switching of haplotype activity to TAC > CGG, in agreement with the stronger binding motif of the TAC haplotype compared to the CGG haplotype (difference 26%, *p* = 0.004).

In summary, these results confirm that the *GCKR* intronic region containing rs780094, rs780095, and rs780096 is able to enhance transcription in a haplotype-specific way. Furthermore, our data support the notion that FOXA2 plays an important role in priming the enhancer for activity and suggest that the collaborative binding between TFs such as MAFK may determine the direction of the haplotype bias in response to specific cellular signals.

### Haplotype-specific H3K27Ac levels and FOXA2 binding

HepG2 cells are heterozygous for rs780094, rs780095, and rs780096, which allows investigation of the haplotype-specific features of this region in a native chromatin context. However, HepG2 cells have also been reported to have a hyper-diploid karyotype with chromosome 2 gain [33]. We found that this gain results in a duplication of the CGG haplotype, which was taken into account in ChIP-Seq data normalization (Additional file 1: Figure S2). ChIP-seq data on HepG2 cells from the ENCODE Project suggest differential histone marks (Additional file 1: Figure S3a) and TF binding (Additional file 1: Figure S3a, b) between the rs780094 alleles. The C allele shows higher H3K4me1 and H3K4me2 histone mark levels as well as higher binding of P300, FOXA2, and FOXA1 TFs. Consistent with the PWM for rs780094 (Fig. 1b) and the reporter assay results (Fig. 2d), the T allele is suggested to have higher MAFK binding (Additional file 1: Figure S3b). Furthermore, a recent study [34] investigating enhancer SNPs capable of disrupting enhancer activity upon allelic change in HepG2 cells identified rs780094 as a dubbed deleterious enhancer (deSNP) with a high risk to cause phenotypic changes (scored 64.07 and ranked in the top 0.25% among all the tested HepG2 enhancer SNPs using unmasked genomic sequences; personal communication from Di Huang and Ivan Ovcharenko). The authors also provide evidence that deSNPs are more likely to display significant allele-specific binding, with 3.7% of H3K4me1-marked loci, 6.2% of H3K27ac-marked enhancers, 25.8% of FOXA2, 20.4% of FOXA1, and 16.5% of P300 binding sites displaying differential enrichment [34]. These results suggest that although the phenomenon of allele bias we detected for rs780094 is a rather rare event based on histone modifications (~5%), genetic variations affecting the recruitment of FOXA transcription factors affect a much larger fraction of enhancers in liver, suggesting an overall stronger impact on regulatory activity [34].

To confirm the ChIP-Seq results, we performed ChIP-qPCR experiments to determine the haplotype-specific levels of the H3K27Ac histone mark and FOXA2 binding in our batch of HepG2 cells using a custom TaqMan Genotyping Assay for rs780094. Although the genotyping assay amplifies a 70-bp region around rs780094, the resolution of the ChIP methods (~250 bp) is not sufficient to discriminate whether the differential binding is allele-specific only for rs780094, thus limiting our analysis to the haplotypes. In line with the active histone marks H3K4me1/2, the CGG haplotype also exhibited 1.4-fold higher enrichment of H3K27Ac (*p* = 0.001; Fig. 3a). Likewise, the CGG haplotype was shown to bind two times more endogenous FOXA2 than the TCC haplotype (Additional file 1: Figure S4). Upon overexpression of FOXA2, the binding of the CGG haplotype was further increased by 2.5-fold compared to the TAC haplotype (*p* = 0.01; Fig. 3b). Fluorescence imaging, western blotting, and qPCR verified the efficiency of transfection and the expression levels of FOXA2 in HepG2 cells (Additional file 1: Figure S5 and S6).

HepG2 cells become insulin resistant when cultured at high glucose [30–32]. However, they respond to acute treatment with insulin independent of glucose concentration, as indicated by substrate phosphorylation and cellular translocation of transcription factors [28, 35, 36]. Hence, we used HepG2 to investigate the specificity of FOXA2 binding between the haplotypes. Insulin stimulation led to a 2.3-fold reduction in the binding of the CGG haplotype to FOXA2 (*p* = 0.002; Fig. 3b), while there was also a trend towards a decrease in the binding of the TAC haplotype (Fig. 3b). These results are consistent with previous reports showing a reduction of FOXA2 binding to chromatin in response to insulin [37]. Taken together, these results confirm that the locus represented by the GWAS rs780094 lead SNP exhibits haplotype bias in histone marks and TF binding.

### CRISPR-dCas9-VPR-mediated activation of the enhancer locus regulates transcription of endogenous *GCKR* in HepG2 cells

Recent evidence suggests that a significant number of enhancers do not regulate the nearest gene [38–40]. To investigate whether the enhancer indeed regulates *GCKR* expression, we targeted the CRISPR-dCas9-VPR (VPR) activator system to this locus [17]. Co-transfection of VPR with two guide-RNA (gRNA) plasmids [18] (VPR + gR1; VPR + gR2) targeted to regions common to both haplotypes (Additional file 1: Figure S7; Additional file 1: Table S1) in HepG2 cells increased total *GCKR* transcription by 1.4-fold, compared to a control gRNA plasmid without targeting gRNA (VPR + gEm) (*p* = 0.0001 for both VPR + gR1 and VPR + gR2; Fig. 4a). To verify that the increase in gene expression was a specific effect of the VPR activator, we also measured epigenetic changes at the targeted site. To this end, VPR + gR1 significantly increased the levels of the enhancer-associated H3K27Ac histone mark in both haplotypes compared to VPR + gEm (2.1-fold for CGG, *p* = 0.002; 2.4-fold for TAC, *p* = 0.0001; Fig. 4b; for better visualization of variations between the experiments see Additional file 1: Figure S8). Given that the CRISPR-targeted regions were identical between the haplotypes, we did not look at the differences between haplotypes upon activation with VPR + gR1. Nevertheless, in agreement with the haplotype bias in basal conditions (Fig. 3a), the CGG haplotype showed higher levels of H3K27Ac in the control, non-targeting VPR + gEm (CGG > TAC, *p* = 0.03; Fig. 4b). These results provide direct evidence that *GCKR* is a target gene for the enhancer.

### Haplotype-specific expression in human liver biopsies

Having demonstrated that the enhancer regulates *GCKR* transcription, we investigated *GCKR* haplotype-specific expression in human liver biopsies from subjects that participated in the Kuopio Obesity Surgery (KOBS) study [20] (Additional file 1: Table S2). To determine whether the CGG and TAC haplotypes were expressed differentially, we selected 132 subjects heterozygous for both haplotypes and measured the haplotype expression levels with a Taqman genotyping assay for rs780094. This strategy allows measurement of the differences in haplotype expression within each sample. Our results indicated that the expression of the CGG haplotype was 18% higher than that of the TAC haplotype (*p* = 2.92 × 10^−4^; Fig. 5a). Moreover, when stratified by sex, we found that the haplotype differences were larger in men than in women (CGG > TAC; men 29%, *p* = 4.24 × 10^−5^, n = 44; women 12%, *p* = 0.051, n = 88; Fig. 5b). The haplotype differences were statistically significant in individuals with normal glucose tolerance (NGT; 18%, n = 75, *p* = 0.001) but only a non-significant trend was observed in individuals with T2D (17%, n = 57, *p* = 0.68) (Additional file 1: Figure S9). Additionally, we assessed the associations of the haplotype expression with metabolic traits in individuals with NGT. We found that higher expression of the CGG haplotype was associated with higher levels of fasting free fatty acids (FFAs; 0.454; *p* = 0.003; *p-adj* = 0.02, Bonferroni correction; n = 42) in individuals with NGT (Additional file 1: Table S3). These results demonstrate that the CGG and TAC haplotypes were differentially expressed in the liver and suggest that the haplotype-specific regulation of this locus affects metabolic outcomes.

## Discussion

More than 100 common variants associated with T2D have been identified by GWAS [1]. However, the translation of these associations into molecular mechanisms has been limited. The vast majority of T2D GWAS variants map to the non-coding regions and recent studies suggest that they mainly function by altering the recruitment of transcription factors and thus leading to variable histone modifications [12, 41]. Identification of the genome-wide DNA regulatory elements by consortiums such as ENCODE [22] and FANTOM [42] has lately made it possible to explore the regulatory potential of variant loci identified by GWAS. In our study we investigated the functional role of a T2D-associated *GCKR* intronic locus represented by the lead SNP rs780094 and proximal variants rs780095 and rs780096. We demonstrate the existence of a transcriptional enhancer in a region containing these three common SNPs that is regulated by FOXA2 and able to activate *GCKR* expression. Moreover, we provide evidence for the haplotype-specific regulation of this region, which might contribute to the related metabolic traits and to the susceptibility for T2D.

**Fig. 6:**
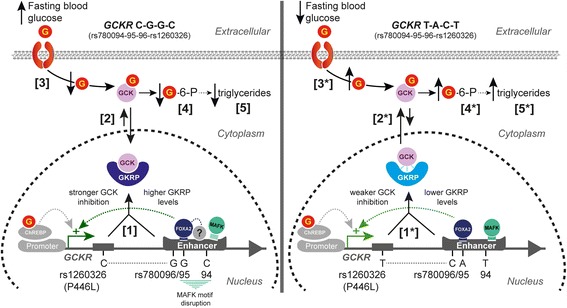
A model for a combined effect of functional haplotypes in *GCKR* on glucose metabolism in fasting. The model assumes higher *GCKR* expression for the CGG haplotype (rs780094-rs780095-rs780096), which is supported by the bias in the transcriptional activity of the haplotypes in response to FOXA2, the H3K27Ac and FOXA2 binding, and the *GCKR* expression in liver (CGG > TAC). The two most common haplotypes formed by rs780094, rs780095, rs780096, and rs1260326 variants in European populations are CGGC (54%) and TACT (40%) (Visualized with LDlink [55]). The CGGC haplotype encodes a GKRP variant with stronger GCK inhibitory capacity in fasting, which combined with higher *GCKR* expression (1) may result in increased nuclear sequestration and decreased cytoplasmic activity of GCK (2). In turn, reduced GCK activity results in reduced glucose uptake and higher blood glucose levels (3) and decreased glucose phosphorylation and disposal (4), which in turn results in lower production of the precursors for the synthesis of triglycerides (5). FOXA2 effects on CGGC haplotype expression may be influenced by the interaction with other TFs (represented by a *question mark*). The TACT haplotype encodes a GKRP protein (P446L) with weaker GCK inhibitory capacity, which combined with lower *GCKR* expression may result in the opposite effects on GCK activity and glucose metabolism (1*, 2*, 3*, 4*, and 5*). The disruption of MAFK motifs by rs780094-C and rs780095-G may change the haplotype bias in *GCKR* transcription upon MAFK activating signals

First, we investigated the enhancer activity of the locus by luciferase reporter assays and ChIP. The associations of this locus with several metabolic traits and the risk of T2D have been previously reported for rs780094 [5, 8, 43–46]. However, the high LD and the proximity of rs780095 and rs780096 SNPs suggest that the potential functional effects might be affected by more than one variant in a haplotype-dependent way. Therefore, we selected the two most common haplotypes formed by rs780094, rs780095, and rs780096 (CGG/TAC) found in the METSIM study [11] for our transcriptional experiments. Our results show that the CGG haplotype, containing the T2D risk-increasing glucose rs780094-C allele, exhibited a higher level of transcriptional activity and higher enrichment for active chromatin marks. The transcriptional function of this site is supported by the observation that epigenetic activation of the native locus using the CRISPR-dCas9-VPR activator [17, 18] leads to an increase in total *GCKR* expression, which also suggests that *GCKR* is a direct target of the enhancer. Interestingly, of all the TFs tested in the luciferase experiments, only FOXA2 was able to activate the regulatory region in a haplotype-specific way (CGG > TAC). The pioneering function of FOXA2 may explain the role of this TF in activating the enhancer. Namely, FOXA2 has the ability to interact directly with the core histones to open condensed chromatin and facilitate the binding of other TFs that would otherwise not be able to access their binding sites [47, 48]. In agreement with the reported inactivating effect of insulin on FOXA2 [27, 28], we showed that insulin reduced FOXA2-dependent transcriptional activity of the enhancer in MPHs, which could be due, at least in part, to the reduction in FOXA2 binding to DNA upon insulin stimulation. Moreover, previous reports have shown that *GCKR* transcription is activated in the fed state by high glucose via ChREBP [49, 50], whereas FOXA2 is activated by glucagon in the fasting state [37]. Thus, our results suggest a novel mechanism for the control of *GCKR* transcription through a FOXA2-activated enhancer in a haplotype-specific way, which may also be contributed to by other TFs (Fig. 6). The relationship between glucagon, FOXA2, and the enhancer in the control of *GCKR* transcription warrants further investigations.

The analysis of haplotype-containing TF motifs identified a MAFK motif disruption at rs780094 and rs780095 loci, which translated to decreased binding of MAFK to the rs780094-C allele in ChIP-Seq. Interestingly, co-expression of FOXA2 with MAFK was able to reverse the transcriptional haplotype bias observed for FOXA2 (TAC > CGG), supporting a co-regulatory role of MAFK [51]. Like FOXA2, MAFK can directly bind DNA to facilitate the formation of transcriptional complexes in partnership with other proteins. A distinctive characteristic of small MAFs, in which MAFK is included, is the lack of a transcriptional activator domain, where the interacting partners and the nuclear concentration of MAFs dictate the direction of regulation (activation/repression) [51, 52]. In light of this knowledge, we can speculate that MAFK forms an interface for other transcriptional regulators to bind and control the activity of the enhancer. Altogether, this supports the current notion that collaborative binding between TFs [12] could impact the overall activity of the enhancer and the functional bias between haplotypes. Further studies are needed to address the specific cellular signals that converge in the activation of the enhancer through the network of TFs.

We also showed that the CGG haplotype exhibited higher expression compared to the TAC haplotype in human liver biopsy samples, providing evidence for a haplotype-specific effect on *GCKR* expression. Moreover, although the differences between the haplotypes were in the same direction, these were of higher magnitude and reached significance only in male subjects, which may have important functional relevance at the population level. Interestingly, the differences in haplotype expression followed the same trend in individuals with NGT and T2D. To our knowledge, changes in *GCKR* expression in T2D have not been reported. Therefore, our data suggest that the genotype may have a stronger effect on *GCKR* expression than the glycemic status. The effect of *GCKR* levels on the diabetic phenotype has been suggested by a study in which overexpression of GKRP in diabetic mice improved glucose homeostasis and increased insulin sensitivity [4]. Further studies are needed to investigate whether the haplotypes are regulated differently in T2D or whether they contribute differentially to the pathophysiology of the disease.

The overall effect of this enhancer on GCKR transcription as well as the haplotype bias are modest, which raises the question of whether such an effect is physiologically relevant. One possible answer to this question may lie in the key role that GKRP plays in controlling GCK availability and activity in the liver by a direct effect on the enzyme kinetics or sequestering it in the nucleus. Previous reports show that two-fold overexpression of rat GKRP is sufficient to reduce GCK translocation, affecting glucose phosphorylation, glycolysis, and glycogen synthesis [4]. Moreover, human GKRP is reported to be a more potent GCK inhibitor than its rat homolog [6, 7], implying that the human GKRP–GCK system may be more sensitive to changes at the level of either GCK or GKRP. Therefore, as suggested previously [8, 50, 53], minor changes in *GCKR* transcription may alter the GCK/GKRP ratio, affect GCK activity, and impact glucose metabolism (Fig. 6). Another possibility is that multiple variants within the *GCKR* locus together contribute to phenotypic traits. Genetic fine mapping of a large LD region on chromosome 2 has identified the non-synonymous rs1260326 SNP as the strongest signal for blood glucose and triglyceride levels in this GWAS locus [5]. Functional studies on this variant demonstrated that the P446L amino acid substitution (rs1260326-T allele) had lower GCK sequestration capacity and lower response to fructose-6-P, affecting the kinetics and cellular localization of GCK [6, 7]. Therefore, it is possible that rs1260326 and the haplotypes formed by rs780094-rs780095-rs780096 account for a combined effect on GCK regulation by changing the inhibitory capacity of GKRP and affecting the level of *GCKR* transcription, respectively (Fig. 6). The involvement of several mechanisms may thus amplify subtle functional defects from individual variants and produce a significant phenotypical effect. Last, it is also possible that, in addition to *GCKR*, the transcriptional enhancer regulates the expression of other genes implicated in different but functionally related pathways.

## Conclusions

The active histone modification marks and the TF binding profiles at the T2D-associated *GCKR* intronic region defined by the high LD SNPs rs780094, rs780095, and rs780096 support the role of this region as a transcriptional enhancer. The SNPs within this locus were shown to overlap the binding motif of FOXA2 and disrupt the binding of MAFK transcription factors. Our data further demonstrate that FOXA2 governs the transcriptional activity of this region in a haplotype-specific way. The CGG haplotype, carrying the T2D-risk rs780094-C allele, exhibited higher reporter gene activity and FOXA2 binding compared to the TAC haplotype. However, co-expression of FOXA2 with MAFK inverted the transcriptional activity to favor the rs780094-T allele that allows higher binding of MAFK, suggesting that specific activating signals determine the direction of the haplotype bias. The epigenetic activation of this locus increased endogenous *GCKR* transcription, suggesting that *GCKR* is a direct target gene of the enhancer. We also showed that the CGG haplotype exhibited higher expression than the TAC haplotype in human liver biopsies, which correlated with higher levels of FFA. Our study reveals for the first time a functional mechanism for a T2D-associated locus represented by the lead SNP rs780094 and including the high LD variants rs780095 and rs780096 that might help in translating the phenotypical associations.

## Additional files

Additional file 1: Supplementary Figures S1–S9 and Supplementary Tables S1–S3. (DOCX 1937 kb)

Additional file 2: Datasets. (XLSX 178 kb)

Additional file 3: Histone modifications at rs780094 from different tissues (data from Roadmap Epigenomics Project). (XLSX 17 kb)

Additional file 4: Raw sequencing data from HepG2, first read. (ZIP 436 kb)

Additional file 5: Raw sequencing data from HepG2, second read. (ZIP 470 kb)

## Abbreviations

ChIP-qPCR: Chromatin immunoprecipitation followed by quantitative PCR
FBS: Fetal bovine serum
FFA: Free fatty acid
gDNA: Genomic DNA
GWAS: Genome-wide association study
IGV: Integrative Genomics Viewer
LD: Linkage disequilibrium
METSIM: Metabolic Syndrome In Men
MPH: Mouse primary hepatocyte
NGT: Normal glucose tolerance
OGTT: Oral glucose tolerance test
PWM: Position weight matrix
SNP: single nucleotide polymorphism
T2D: Type 2 diabetes
TF: Transcription factor.
