# Supplementary material for: Identification and characterization of a FOXA2-regulated transcriptional enhancer at a type 2 diabetes intronic locus that controls GCKR expression in liver cells
Source: Genome Med. 2017 Jul 6;9:63. doi: 10.1186/s13073-017-0453-x (PMC5501007; doi:10.1186/s13073-017-0453-x)
Supplement: Supplementary file 1 — Supplementary Figures S1–S9 and Supplementary Tables S1–S3. (DOCX 1937 kb) [file 13073_2017_453_MOESM1_ESM.docx]

**Additional File 1**

**
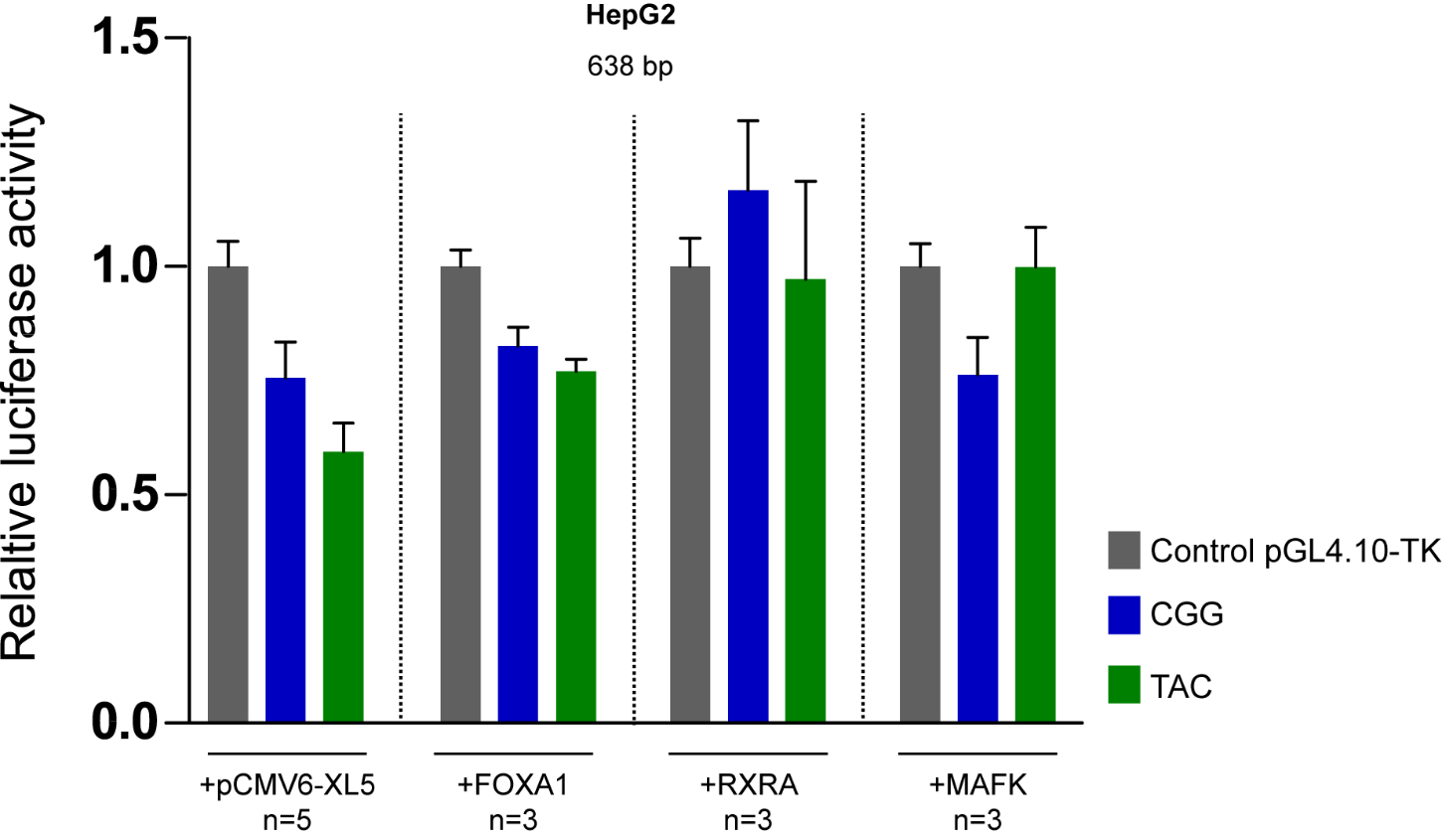
**

Figure S1. **Haplotype-specific transcriptional activity in response FOXA1, RXRA and MAFK.**

Luciferase reporter assays. Similarly to the control “+pCMV6-XL5”, FOXA1, RXRA or MAFK, did not induce the transcriptional activity of the 638 bp regions over the control pGL4.10-TK in HepG2 cells. The results are expressed as relative luciferase activity respect to the control vector pGL4.10-TK. Error bars represent the standard deviation of the total of experiments (n). (+pCMV6-XL5: 3, 3, 8, 4 and 4 technical replicates ; +FOXA1, +RXRA, and + MAFK: 4, 4 and 8 technical replicates).


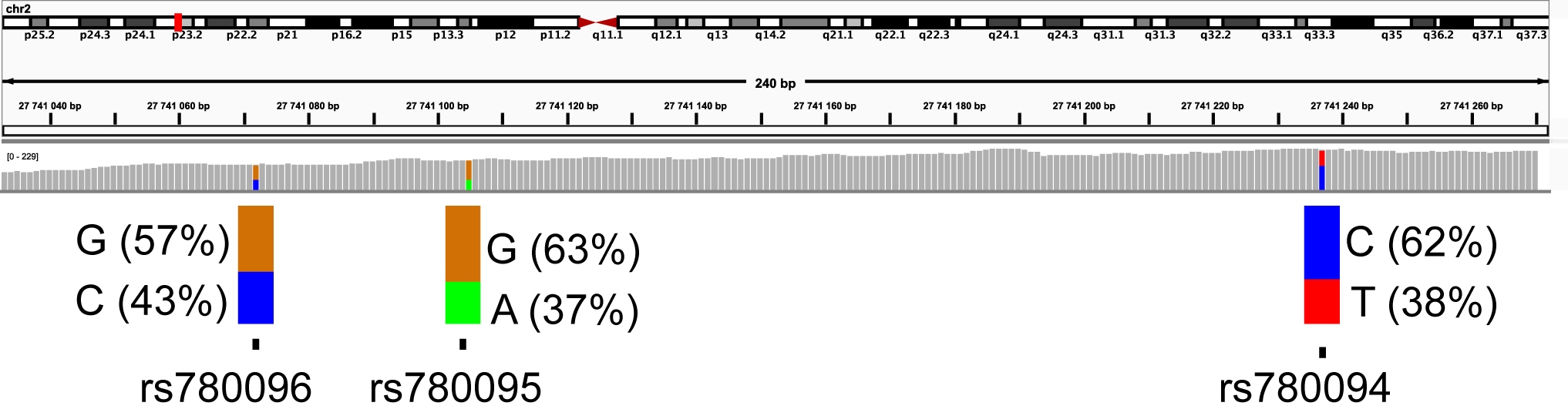


Figure S2. **Allelic distribution for rs780094, rs780095 and rs780096 in HepG2 cells**.

Sequencing data from HepG2 gDNA visualized with IGV genomic viewer. A 3 kb genomic-RNA region around rs780094 was amplified by PCR, prepared with the Nextera XT DNA Library Preparation Kit and ran in a MiSeq desktop sequencer (Illumina). The dual-colored boxes represent actual allelic distribution for each SNP. The CGG (rs780094, rs780095, rs78096) is roughly duplicated compared to TAC.

**
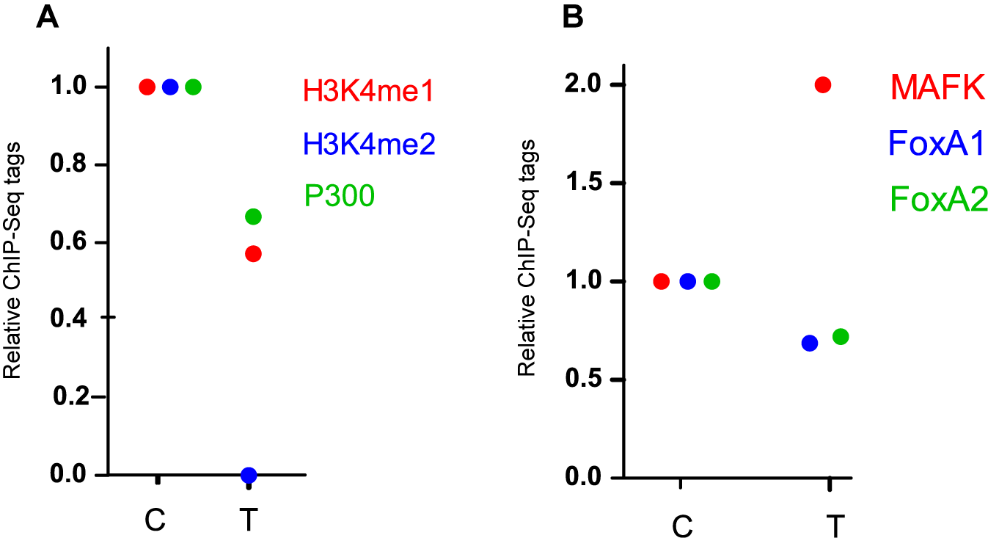
**

Figure S3. **ENCODE histone modification and TF binding ChIP-Seq tags at rs780094**

Relative ChIP-seq tags for the rs780094-T allele relative to rs780094-C allele obtained from the ENCODE project, and normalized to the allelic imbalance resulting from the hyperdyploid kariotype of HepG2 cells (C=2XT). **a** H3K4me1, H3K4me2 and P300 enhancer marks tags. **b** MAFK, FOXA1 and FOXA2 TFs binding.


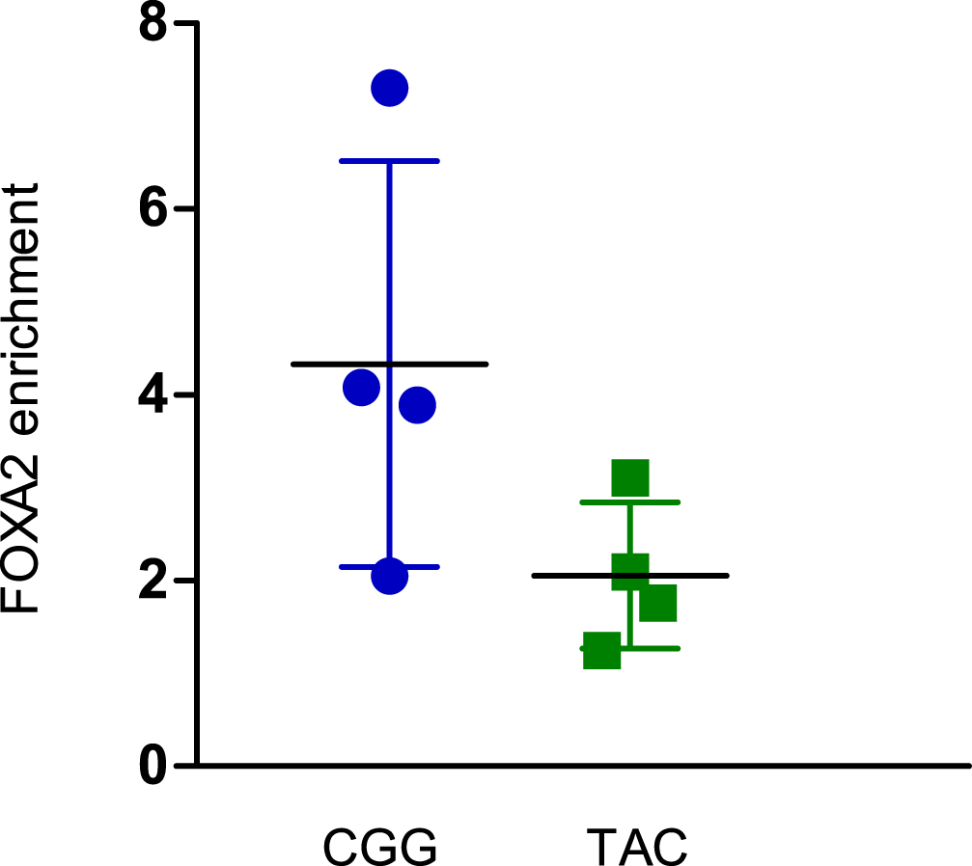


Figure S4. **Haplotype specific binding of Foxa2.**

HepG2 endogenous Foxa2 ChIP-qPCR. Chromatin was immunoprecipitated and the allele specific binding was determined by qPCR using custom TaqMan SNP Genotyping Assay for rs780094.The data represents haplotype-specific enrichment over the input normalized to a region of GRB10 with no TF binding (mean ± SD). Two experiments] with two independent replicates each were performed [n=4].

**n=4**


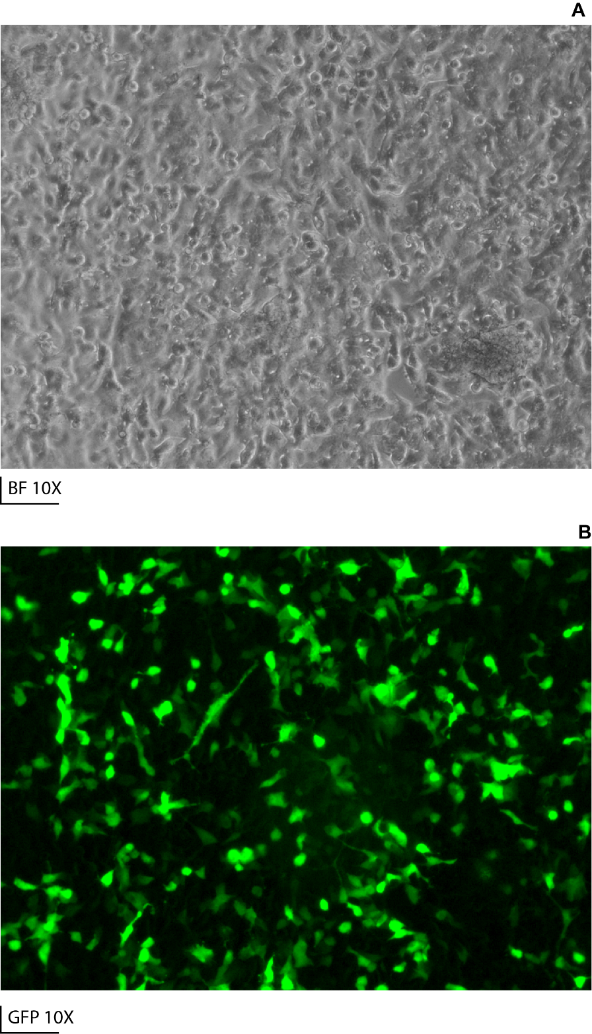


Figure S5. **Verification of transfection efficiency in HepG2 cells transfected with a plasmid expressing a green fluorescent protein (GFP)**.

**a** Bright field (BF) image. **b** GFP fluorescence image (same field). Efficiency of transfection: about 80%. (10X magnification).

**
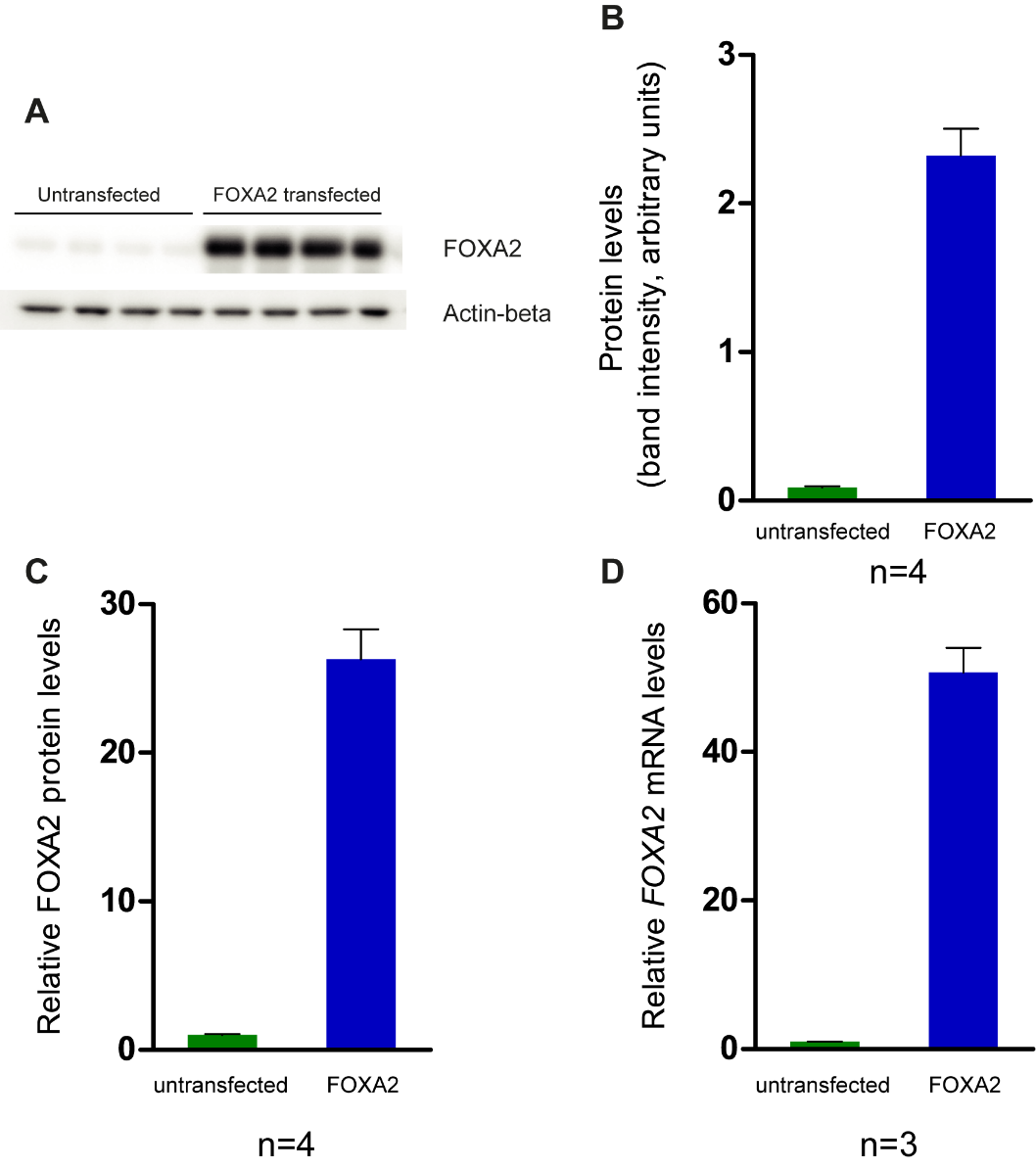
**

Figure S6. **Efficiency of expression of pCMV-XL5-FOXA2 plasmid in HepG2 cells.**

**a** FOXA2 western-blot. **b, c** Protein quantification. **d** Relative *FOXA2* mRNA expression. (anti-FOXA2 antibody: AB_2262810; mRNA detection by Taqman qPCR).

**
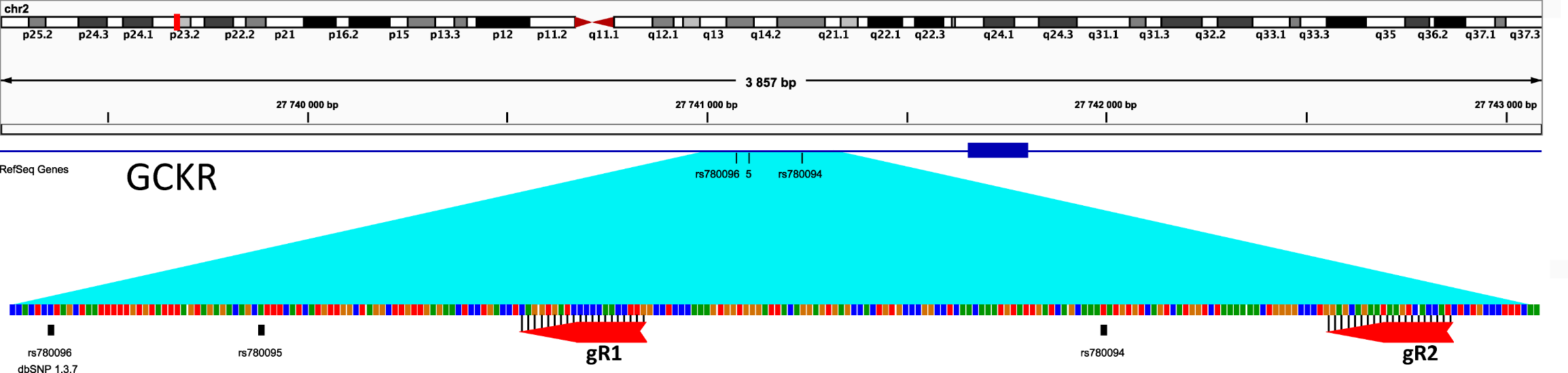
**

Figure S7**. Schematic representation of the CRISPR guide-RNA locations**

Guide-RNAs gR1 and gR2 target to SNP free regions, thus identical between the CGG and TAC haplotypes. Both gR1 and gR2 target the minus strand. SNP location is visualized with IGV genomic viewer.

**
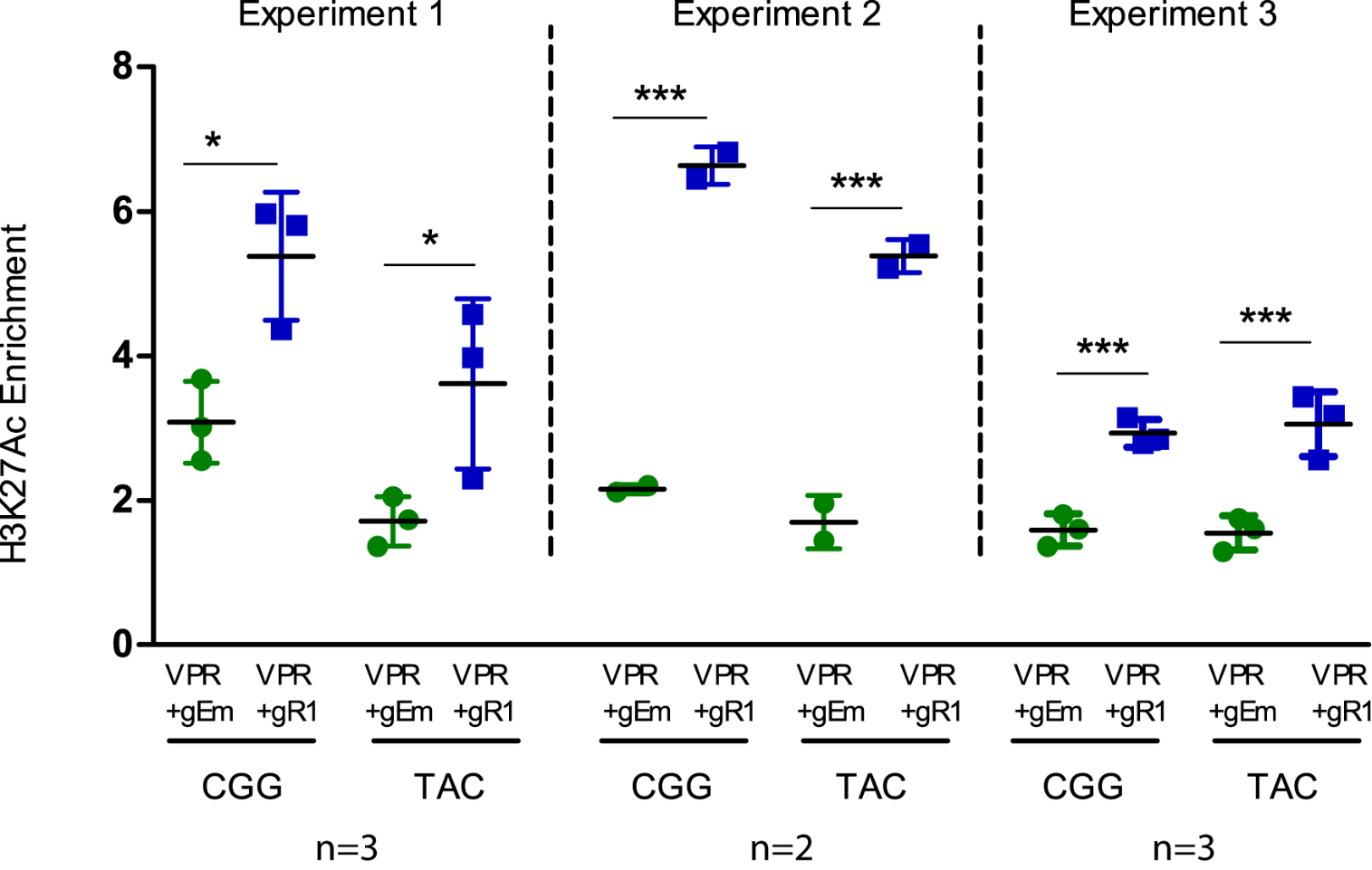
**

Figure S8. **H3K27Ac enrichment at rs780094 locus induced by CRISPR-dCas9-VPR in three independent experiments.**

HepG2 cells were co-transfected with the VPR activator plasmid and the targeting gR1 or the non-targeting gEm gRNA plasmids. Haplotype specific enrichment of H3K27Ac (enrichment over input normalized to a region of *GRB10* with no TF binding [rs6943153]) was determined by ChIP-qPCR using the custom Taqman SNP Genotyping Assay for rs780094. Error bars represent the standard deviation for 3 technical replicates in each experiment [n=3]. Stars depict statistical significance (*p≤0.05; **p≤0.01; ***p≤0.005; two-tailed *t*.test).

**
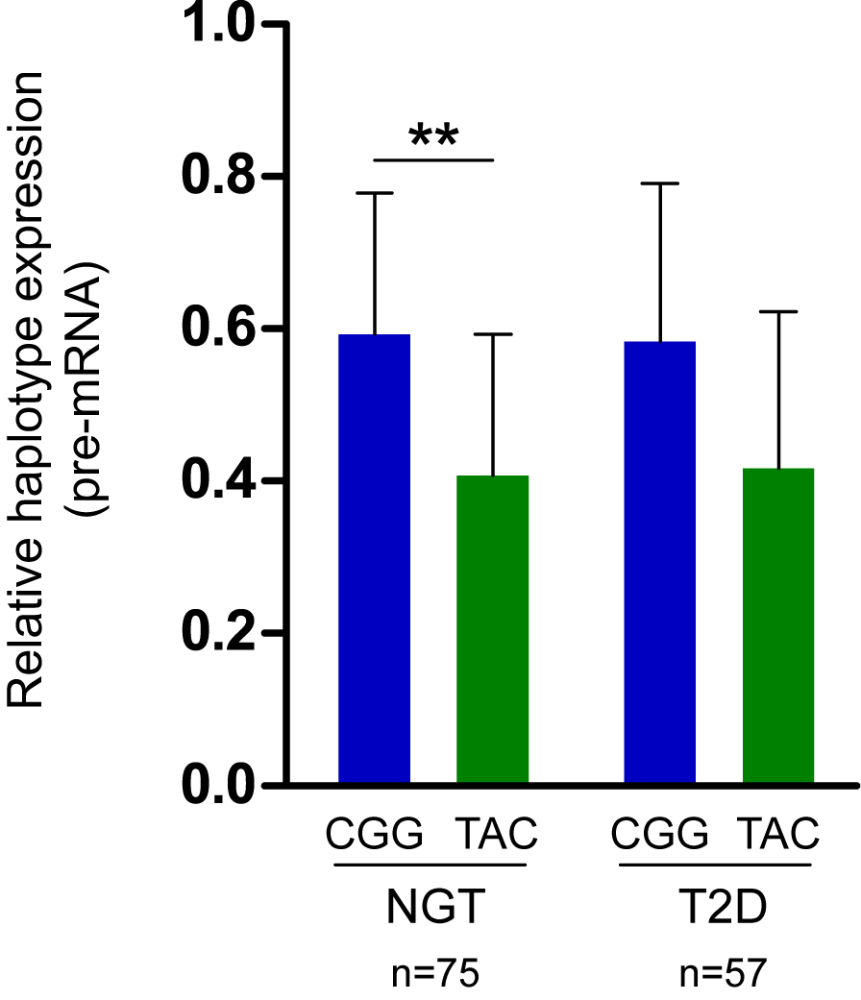
**

Figure S9. **Haplotype specific expression in human liver biopsies stratified by glycemic status.**

Haplotype levels of pre-mRNA expression determined with the TaqMan SNP Genotyping Assay for rs780094. The data is presented separately for NGT (n=75) and T2D (n=57) individuals. The allelic expression bias is statistically significant for NGT individuals and a similar trend is found got T2D. The data represent the mean expression of each allele relative to the other. Error bars represent the standard deviation and the stars depict statistical significance (**p≤0.01; Mann–Whitney *U* test)

| **Table S1. DNA primers and oligoes**  The ID, the sequence and the specific application within the study are indicated | | | |
| --- | --- | --- | --- |
|  |  |  | |
| **Primer/oligo ID** | **Sequence** | **Application** | |
| - Fw-BamHI-600  - Rev-SalI-600 | - aaaggatccgggttcaagtgattcttgtgcc  - aaagtcgacagacacatgggggtgtcaaaaa | For cloning the 620 bp fragments of GCKR into luciferase vector. |  |
| - Fw-BamHI-3Kb  - Rev-SalI-3Kb | - aaaggatccttacctccacctggtctctct  - aaagtcgactcctcagtcacattagccaca | - For cloning the 3 Kb fragments of GCKR into luciferase vector.  - For PCR amplification before sequencing. |  |
| - FW-rs780094-TC  - Rev-rs780094-TC  - rs780094-TCM2  - rs780094-TCV2 | - cccggcctcaacaaatgtattg  - ctccagggccccagtttt  - FAM-tgacacatgtttgctg-NFQ  - VIC-actgacacatatttgctg-NFQ | Components of the Taqman SNP Genotyping Assay for rs780094 (C/T). Both Fam and Vic conjugated probes are in the reverse orientation. |  |
| - Fw-rs6943153-AG  -Rev- rs6943153-AG  - rsrs6943153-AGM  - rs6943153-AGV | - tgtgggtccctctgtagct  - caaggcaaacagacataggaaggt  - FAM-agtgtgacctgcctcc-NFQ  - VIC-cagtgtgacctacctcc-NFQ | Components of the Taqman SNP Genotyping Assay for rs6943153 (A/G). Both Fam and Vic conjugated probes are in the forward orientation. |  |
| - Fw-gR1  - Rev-gR1  - Fw-gR2  - Rev-gR2 | - caccgatggtgacttattctgctcc  - aaacggagcagaataagtcaccatc  - caccgtcaatacatttgttgaggcc  - aaacggcctcaacaaatgtattgac | For gRNA double stranded oligoes preparation and sub-cloning into pSPgRNA plasmid. |  |

| **Table S2. Characteristics of the subjects heterozygous for all 3 SNPs (rs780094, rs780095, rs780096) allele identified from the KOBS study** | | | |
| --- | --- | --- | --- |
|  |  |  |  |
| **Feature/trait** | | **Mean** | **Std. Deviation** |
| Male/Female | | 44/88 |  |
| Age (years) | | 47.85 | 9.17 |
| BMI (kg/m^2^) | | 43.51 | 5.78 |
| Fasting glucose (mmol/L) | | 6.67 | 1.89 |
| Fasting insulin (pmol/L) | | 155.55 | 223.77 |
| Triglycerides (mmmol/l) | | 1.66 | 0.80 |
| Total cholesterol (mmol/l) | | 4.22 | 0.90 |
| HDL cholesterol (mmol/l) | | 1.12 | 0.33 |
| Fasting FFA (mol/l) | | 0.66 | 0.25 |
| Type 2 diabetes | | 57 |  |

| **Table S3. Correlations between haplotype expression and metabolic traits in heterozygous normo-glycemic subjects from the KOBS study.** Fasting FFAs correlate with allelic expression (Spearman's test). BMI, body mass index; FFA, free fatty acids. | | | | | | | | | | | | | | | | | | | |
| --- | --- | --- | --- | --- | --- | --- | --- | --- | --- | --- | --- | --- | --- | --- | --- | --- | --- | --- | --- |
|  | | | | | | | | | | | | | | | | | | | |
| **rs780094 rs780095 rs780096** |  | | | **Age (years)** | | **BMI (kg/m2)** | | **Fasting glucose (mmol/l)** | | **Fasting insulin (pmol/l)** | | **Total cholesterol (mmol/l)** | | **HDL cholesterol (mmol/l)** | | **Triglycer-ides (mmmol/l)** | | **Fasting FFA (mol/l)** | |
| **TAC** | | **r** | -0.099 | | -0.148 | | -0.174 | | 0.068 | | 0.087 | | -0.047 | | 0.024 | | **-0.454** | |  |
|  | | **p** | 0.399 | | 0.204 | | 0.139 | | 0.565 | | 0.463 | | 0.693 | | 0.838 | | **0.003** | |  |
|  | | \| **p-adj** \| 1 \| 0.872 \| 0.336 \| 1 \| 1 \| 1 \| 1 \| **5.9 × 10^-4^** \| \| --- \| --- \| --- \| --- \| --- \| --- \| --- \| --- \| --- \| | 1 | | 1 | | 1 | | 1 | | 1 | | 1 | | 1 | | **0.020** | |  |
|  | | **N** | 75 | | 75 | | 74 | | 74 | | 73 | | 73 | | 73 | | **42** | |  |
| **CGG** | | **r** | 0.099 | | 0.148 | | 0.174 | | -0.068 | | -0.087 | | 0.047 | | -0.024 | | **0.454** | |  |
|  | | **p** | 0.399 | | 0.204 | | 0.139 | | 0.565 | | 0.463 | | 0.693 | | 0.838 | | **0.003** | |  |
|  | | **p-adj** | 1 | | 1 | | 1 | | 1 | | 1 | | 1 | | 1 | | **0.020** | |  |
|  | | **N** | 75 | | 75 | | 74 | | 74 | | 73 | | 73 | | 73 | | **42** | |  |
